# Signal Processing Method and Evaluation Method for Weak Doppler Signal Detection

**DOI:** 10.3390/s25123775

**Published:** 2025-06-17

**Authors:** Chongbin Xi, Jian Zhou, Xiaoming Nie, Shilong Jin

**Affiliations:** 1 College of Advanced Interdisciplinary Studies, National University of Defense Technology, Changsha 410073, China; xichongbin@nudt.edu.cn (C.X.); nxm@nudt.edu.cn (X.N.); sljin@nudt.edu.cn (S.J.); 2 Nanhu Laser Laboratory, National University of Defense Technology, Changsha 410073, China

**Keywords:** laser Doppler velocimeter, weak Doppler signal, signal superposition, relative prominence, threshold of relative prominence

## Abstract

When the amplitude of the laser Doppler signal is less than that of the noise amplitude, the speed information cannot be identified. In this paper, the recognition of weak Doppler signals is accomplished by superimposing multiple-frame Doppler signals in the frequency domain and eliminating the base noise. Meanwhile, the relative prominence is defined to assess the quality of Doppler signals. The theoretical analysis and experimental results demonstrate that the relative prominence can be enhanced by more than two orders of magnitude through the stacking of 10,000 frames of Doppler signals. Moreover, this paper establishes a foundation for determining the threshold of relative prominence in practical measurements. When the relative prominence at a specific point on the spectrum is 3, there is at least a 99.08% probability that this point corresponds to the target signal.

## 1. Introduction

The Laser Doppler Velocimeter (LDV) receives the scattered light of moving particles and combines it with the reference light to obtain the Doppler signal [1,2]. Upon applying the Fourier transform to the Doppler signal, the signal peak can be discerned from the signal spectrum, and subsequently, the target speed can be computed [1]. The LDV is extensively utilized in the measurement of velocity, vibration, displacement, and other physical quantities [3,4,5] due to its high accuracy, wide range, high spatial resolution, and non-contact measurement. Furthermore, LDV can also achieve fully autonomous, all-day, and all-weather speed measurements, thus being employed in integrated navigation and positioning systems [6,7,8]. In recent years, LDV has been applied in the speed measurement and positioning of unmanned aerial vehicles (UAV) underwater vehicles [9,10,11].

Constrained by the intensity of the scattered light and the size of the receiving aperture, the signal light received by LDV is exceedingly weak. Additionally, when LDV is employed for underwater measurement or UAV speed measurement, the increase in the propagation distance of the laser and the alteration of the transmission medium will augment the loss of signal light, which will further attenuate the signal strength, and the spectrum of the Doppler signal is submerged within the noise spectrum. Hence, it is necessary to utilize a high-power light source, increasing the volume and power consumption of the system. This is also a major factor restricting the use of LDV for underwater and airborne carrier measurements. Traditional pulsed lidar or frequency-modulated continuous-wave lidar can effectively achieve time-domain superposition of Doppler signals through precise phase alignment [12]. However, for non-frequency-modulated continuous-wave LDV, the phase pulsation inherent in the synthesized Doppler signal leads to suboptimal performance after time-domain superposition and may even result in signal cancelation. In addition, wavelet transform can reduce or even remove noise [13], but this noise may cause some blurring to a certain extent. Consequently, it is of considerable significance to study the signal processing method for weak Doppler signal recognition for the practical application of LDV.

This paper presents a signal processing methodology for the recognition of weak Doppler signals. By contrasting time-domain superposition and frequency-domain superposition, it is deduced that multi-frame Doppler signals are superimposed in the frequency domain, and subsequently, the base noise is eliminated to realize the recognition of signal peaks. Concurrently, the relative prominence is defined to appraise the quality of the Doppler signal. Through theoretical analysis and experimental corroboration, the relative prominence varies with the number of superimposed frames. Additionally, the selection basis of the relative prominence threshold in practical measurement is provided, which holds great significance for the practical application of LDV.

## 2. Theory and Simulation

### 2.1. Principle of Superposition of Doppler Signal

Suppose the Doppler signal is a stationary random signal; the beat frequency signal corresponding to each frame captured by the LDV can be articulated as follows:

(1) $$D\left(t\right)=s\left(t\right)+n\left(t\right)$$

where *s*(t) represents a noise-free signal, *n*(t) is Gaussian white noise, which is independent and uniformly distributed, and $n∼N\left(\mu_{t},\sigma_{t}^{2}\right)$, where *μ_t_* and *σ_t_* are the mean and standard deviation of the time-domain noise, respectively.

The signal spectrum can be derived from the Fourier transform of the Doppler signal, assuming that signal amplitude and noise amplitude are *A*_s_ and *A*_n_, respectively. According to the properties of the Fourier transform, when only the positive half-axis of the spectrum is considered, $FFT(n)∼N(\mu_{t},\sigma_{t}^{2})$. Then, $A_{n}=\left|FFT\left(n\right)\right|$ follows a half-normal distribution. The probability distribution function (PDF) of the half-normal is given by:

(2) $$f\left(A_{n}\right)=\frac{\sqrt{2}}{\sigma_{t}\sqrt{\pi }}\exp\left(-\frac{A_{n}^{2}}{2\sigma_{t}^{2}}\right)$$

where

(3) $$E\left[A_{n}\right]=\frac{\sigma_{t}\sqrt{2}}{\sqrt{\pi }}$$

(4) $$D\left[A_{n}\right]=\sigma_{t}^{2}\left(1-\frac{2}{\pi }\right)$$

The signal-to-noise ratio (SNR) of each frame signal can be expressed as:

(5) $$SNR_{1}=\frac{A_{s}}{E\left(A_{n}\right)}=\frac{A_{s}}{\sigma_{t}}\sqrt{\frac{\pi }{2}}$$

where *A*_s_ is the signal amplitude without noise, and *E*(*A*_n_) is the expectation of noise amplitude. For the convenience of analysis, it is assumed that the Doppler signal is an ideal sinusoidal signal, and the initial phase and amplitude of each frame signal are the same; the superposition of m-frames in the time domain can be expressed as follows:

(6) $$ℜ\left(t\right)=\sum_{i=1}^{m}D_{i}\left(t\right)=\sum_{i=1}^{m}\left[s_{i}\left(t\right)+n_{i}\left(t\right)\right]=\sum_{i=1}^{m}s_{i}\left(t\right)+\sum_{i=1}^{m}n_{i}\left(t\right)=ms+\sum_{i=1}^{m}n_{i}\left(t\right)$$

According to the properties of Gaussian distribution, $\sum_{i=1}^{m}n_{i}\left(t\right)∼N\left(\mu_{t},m\sigma_{t}^{2}\right)$. Consequently, the expected value of noise in the Doppler signal spectrum after the superposition in the time domain of m-frames is

(7) $$E\left(A_{nm}\right)=E\left(\left|FFT\left(\sum_{i=1}^{m}n_{i}\left(t\right)\right)\right|\right)=\frac{\sqrt{m}\sigma_{t}\sqrt{2}}{\sqrt{\pi }}$$

The SNR of the Doppler signal after superposition in the time domain can be expressed as

(8) $$SNR_{n-t}=\frac{mA_{s}}{E\left(A_{nm}\right)}=\frac{mA_{s}}{\sqrt{m}\sigma_{t}}\sqrt{\frac{\pi }{2}}=\sqrt{m}\cdot SNR_{1}$$

It is evident that after the superposition of m-frames of the Doppler signal in the time domain, the SNR can be enhanced by a factor of $\sqrt{m}$.

Doppler signals can be superimposed not only in the time domain but also in the frequency domain. The SNR after superposition in the frequency domain can be expressed as:

(9) $$SNR_{n-f}=\frac{mA_{s}}{mE\left(A_{n}\right)}=\frac{A_{s}}{E\left(A_{n}\right)}=SNR_{1}$$

It can be seen from Equation (9) that the superposition of Doppler signals in the frequency domain does not enhance the SNR. This phenomenon primarily arises because when Doppler signals are superimposed in the frequency domain, the amplitude of the noise spectrum also increases alongside the superimposed spectrum.

### 2.2. Simulation

**Figure 1:**
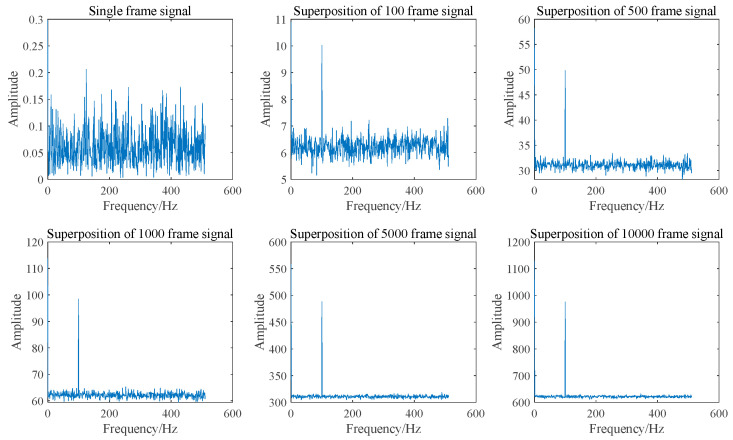
Spectrum diagram of signal superposition in the time domain with different frame numbers.

Assuming that the amplitude of each frame of the Doppler signal remains constant, with identical initial phases and a signal frequency of 100 Hz, the spectrum of Doppler signals with varying frame numbers after superposition in the time domain is illustrated in Figure 1.

**Figure 2:**
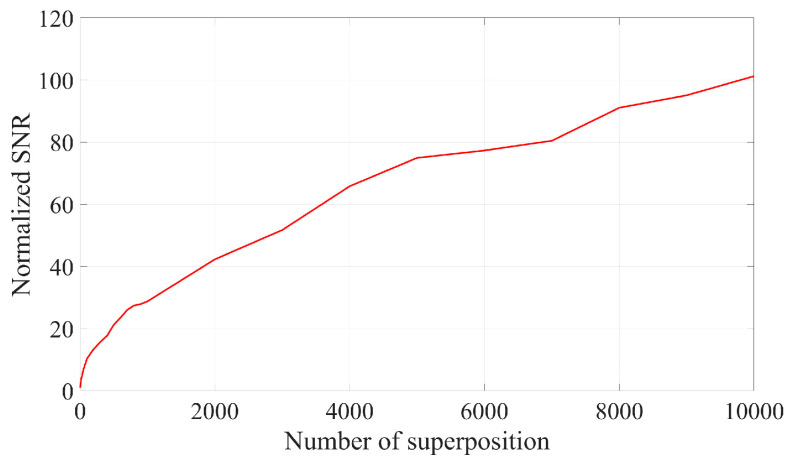
Normalized SNR for time domain superposition of signals with different frame numbers.

The SNR after the superposition of signals with varying frame numbers is presented in Figure 2. The figure indicates that the normalized SNR after the superposition of 10,000 frames is 104.226, which aligns closely with theoretical predictions.

**Figure 3:**
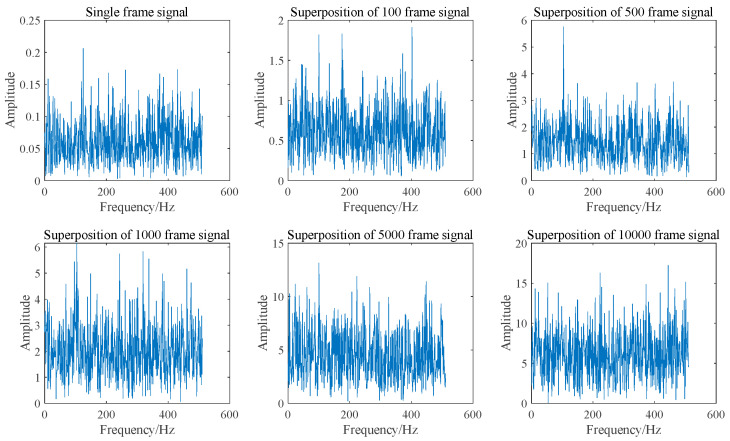
Time domain signal superposition spectra with random initial phase of different frames.

**Figure 4:**
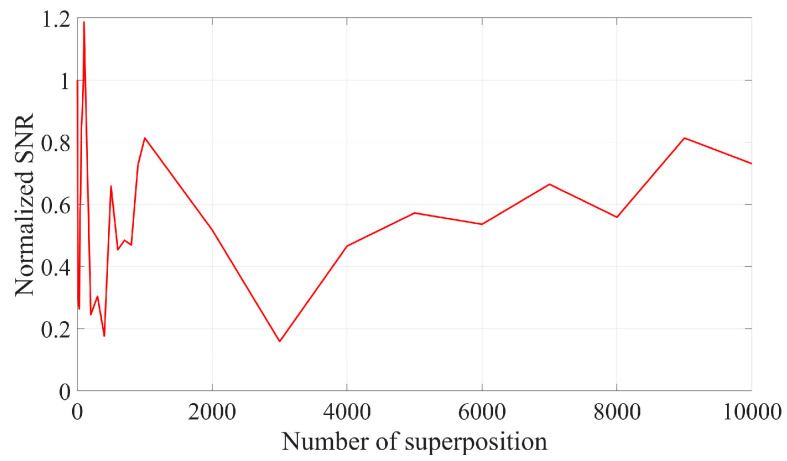
Normalized SNR of time domain superposition of signals with random initial phase of different frames.

As illustrated in Figure 1 and Figure 2, the SNR can be enhanced by two orders of magnitude when 10,000 frames of Doppler signals are superimposed in the time domain. This improvement is attributed to the coherent superposition of Doppler signals across different frames, necessitating that their initial phases remain identical. However, due to the random distribution of scattered particles on the moving surface, the initial phases of Doppler signals from different frames may not be equal. Consequently, the actual effect of superimposing Doppler signals in the time domain is sub-optimal and may even result in signal cancelation. The signal and noise are maintained at the same amplitude; however, each frame of the Doppler signal exhibits a random initial phase. The result of superposition in the time domain is illustrated in Figure 3 and Figure 4. It can be seen that the superposition of the signal in the time domain fails to enhance the SNR, rendering it impossible to identify the Doppler frequency from the signal spectrum.

**Figure 5:**
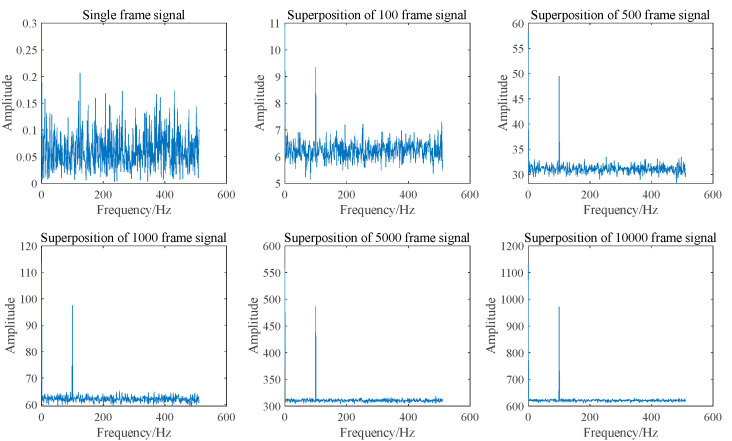
Spectrum diagram of signal superposition in the frequency domain with different frame numbers.

**Figure 6:**
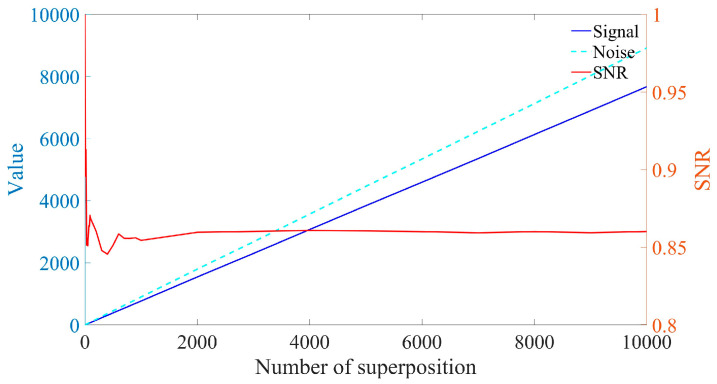
Normalized SNR for frequency domain superposition of signals with different frame numbers.

In addition to superimposing signals in the time domain, they can also be superimposed in the frequency domain. Since the Amplitude-Frequency diagram of a signal does not include phase information, the random initial phase will not impact frequency domain superposition. Figure 5 and Figure 6 illustrate the spectrum and SNR of signals with varying frame numbers that have been superimposed in the frequency domain. As shown in Figure 6, both the signal amplitude and noise amplitude increase with the number of superimposed frames, which aligns with the analysis presented in Section 2.1. The SNR of the signal after superposition in the frequency domain basically remains unchanged, which is consistent with the results obtained in Equation (9).

As illustrated in Figure 5, when signals with varying frame numbers are superimposed in the frequency domain, Doppler peaks can be generated, allowing for the identification of Doppler frequencies. Furthermore, the signal spectra corresponding to different frame numbers exhibit significant differences. However, the SNR presented in Figure 6 remains largely unchanged. This indicates that SNR is not an effective measure of Doppler signal quality. Consequently, a new parameter is required to assess the quality of the Doppler signal.

### 2.3. Evaluation Parameter-Relative Prominence

**Figure 7:**
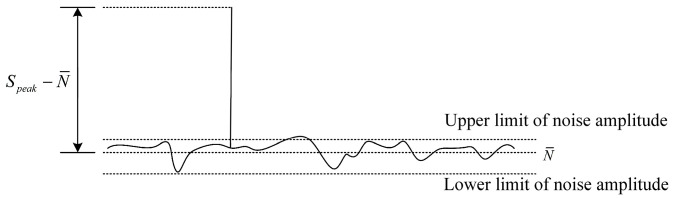
Diagram of relative prominence.

From the Doppler signal spectrum, it can be seen that the prominence of the signal peak relative to the noise will affect whether the signal peak can be recognized. Therefore, relative prominence is proposed to evaluate the signal quality of the Doppler signal in the frequency domain. As shown in Figure 7, relative prominence is defined as the ratio of the fluctuation degree of the Doppler signal peak relative to the mean noise to the fluctuation degree of the noise itself, that is, the standard deviation of the noise. Suppose the signal spectrum value is *N_i_* (*i* = 1, 2, … *M*), the *l*-th point is the location of the signal peak, and the signal peak is Speak. The specific expression is shown in Equation (10).

(10) $$RP=\frac{S_{peak}-\bar{N}}{\sqrt{\frac{\sum_{i=1}^{l-1}{\left(N_{i}\;-\;\bar{N}\right)}^{2}+\sum_{i=l+1}^{M}{\left(N_{i}\;-\;\bar{N}\right)}^{2}}{M\;-\;1}}}$$

where $\bar{N}=\frac{\sum_{i=1}^{l-1}N_{i}+\sum_{i=l+1}^{M}N_{i}}{M-1}$ is the average of noise.

The amplitude of Gaussian white noise after Fourier transformation in exchange for modulus obeys half-normal distribution. According to Equations (3) and (4), the expectation and variance of the mode after Fourier transform of the noise in the Doppler signal are:

(11) $$\mu_{f}=\frac{\sigma_{t}\sqrt{2}}{\sqrt{\pi }}$$

(12) $$\sigma_{f}^{2}=\sigma_{t}^{2}\left(1-\frac{2}{\pi }\right)$$

where the subscript *f* represents the frequency domain. Assuming that the amplitude of the signal in the spectrum of a single-frame Doppler signal is *S*, the relative prominence of a single-frame Doppler signal can be expressed as:

(13) $$RP_{1}=\frac{S-\mu_{f}}{\sigma_{f}}=\frac{S-\sigma_{t}\sqrt{\frac{2}{\pi }}}{\sigma_{t}\sqrt{1-\frac{2}{\pi }}}$$

Since the Gaussian noise in the Doppler signal of different frames is independently and equally distributed, from the central limit theorem, the distribution function satisfies [14,15,16]:

(14) $$\underset{m\to \infty }{\lim}F_{m}\left(x\right)=\underset{m\to \infty }{\lim}P\left\{\frac{\sum_{i=1}^{m}n_{i}-m\mu }{\sigma \sqrt{m}}\leq x\right\}=\frac{1}{\sqrt{2\pi }}\int_{-\infty }^{x}e^{-\frac{t^{2}}{2}}dt=\phi \left(x\right)$$

Equation (14) shows that when m is large, $Y_{m}=\frac{\sum_{i=1}^{m}n_{i}-m\mu_{f}}{\sigma_{f}\sqrt{m}}$ follows the standard normal distribution. Let $N_{m}=\sum_{i=1}^{m}n_{i}$, then *N*_m_ follows a normal distribution, $N_{m}∼N\left(m\mu_{f},m\sigma_{f}^{2}\right)$. Therefore, the relative prominence of the m-frame Doppler signal after superposition in the frequency domain can be expressed as:

(15) $$RP_{m}=\frac{mS-m\mu_{f}}{\sqrt{m}\sigma_{f}}=\sqrt{m}\frac{S-\sigma_{t}\sqrt{\frac{2}{\pi }}}{\sigma_{t}\sqrt{1-\frac{2}{\pi }}}=\sqrt{m}RP_{1}$$

**Figure 8:**
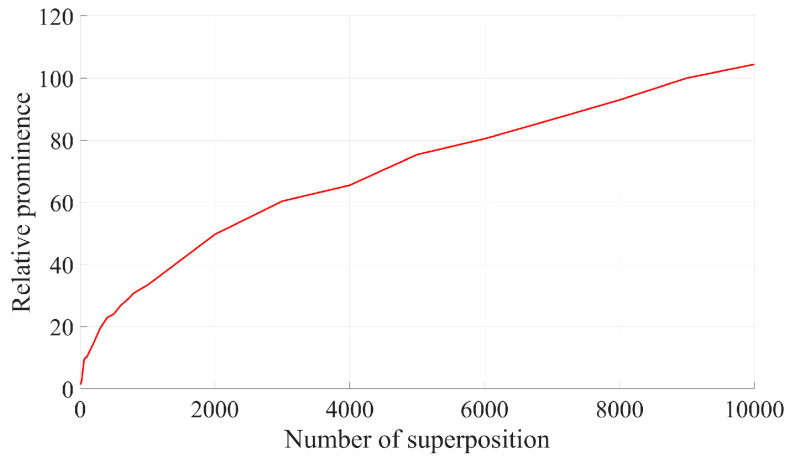
Normalized RP for frequency domain superposition of signals with different frame numbers.

According to Equation (15), the relative prominence of the m-frame Doppler signal after superposition in the frequency domain can be increased by $\sqrt{m}$ times. The relative prominence of Doppler signals after superposition with different frame numbers in the frequency domain in Figure 5 is shown as follows:

As illustrated in Figure 6 and Figure 8, relative prominence effectively represents the quality of Doppler signals after superposition in the frequency domain, in contrast to the SNR.

## 3. Experiment and Discussion

### 3.1. Experimental Result

**Figure 9:**
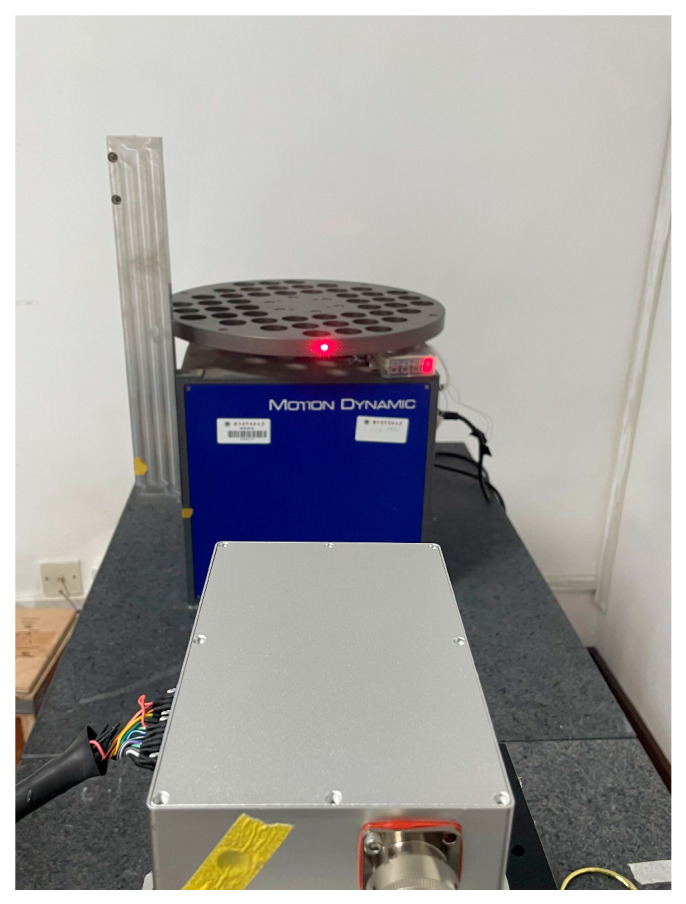
Experimental system.

**Figure 10:**
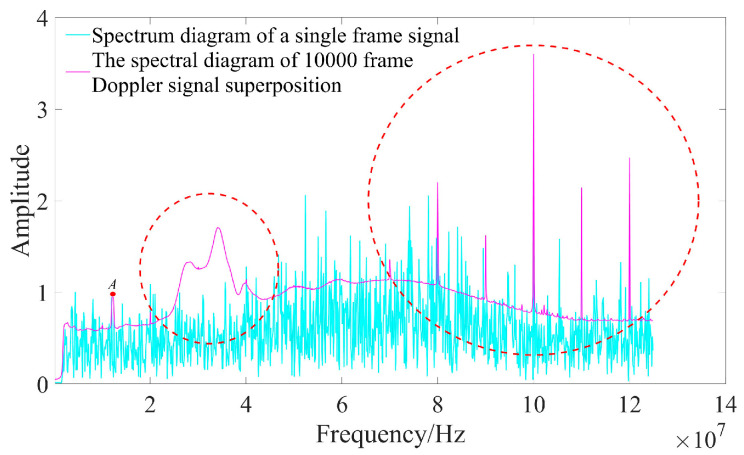
The spectral diagram of 10,000 frame Doppler signal superposition.

The experimental system utilized in this study is depicted in Figure 9, which includes an LDV and a high-precision turntable. Among them, the light source of LDV adopts a 532 nm laser. During the experiment, the speed of the turntable is controlled at intervals of 10 degrees per second, increasing from 10 degrees per second to 1200 degrees per second. In total, 10,000 frames of Doppler signals were collected and subsequently superimposed in the frequency domain. The resulting spectrum from the superimposition of these 10,000 frames is presented in Figure 10.

To ascertain the relative magnitude of the signal peak, the Doppler signal spectrum is normalized against the signal peak value. As illustrated in Figure 10, the spectrum diagram of a single frame signal does not allow for the complete identification of signal peaks. However, when 10,000 frames of Doppler signals are superimposed, the position of the signal peak becomes distinctly visible at point *A* in Figure 10. Concurrently, multiple interference peaks appear on the Doppler signal spectrum diagram, as indicated by the red dotted circle in Figure 10. This phenomenon primarily arises from fixed-frequency noise present within both the circuit system and light source; during the superposition of the Doppler signal spectra, this portion also accumulates. Consequently, if only a straightforward superposition of Doppler signal spectra is performed, accurate identification of the signal peak may be compromised.

### 3.2. Discussion

#### 3.2.1. Removal of Base Noise

**Figure 11:**
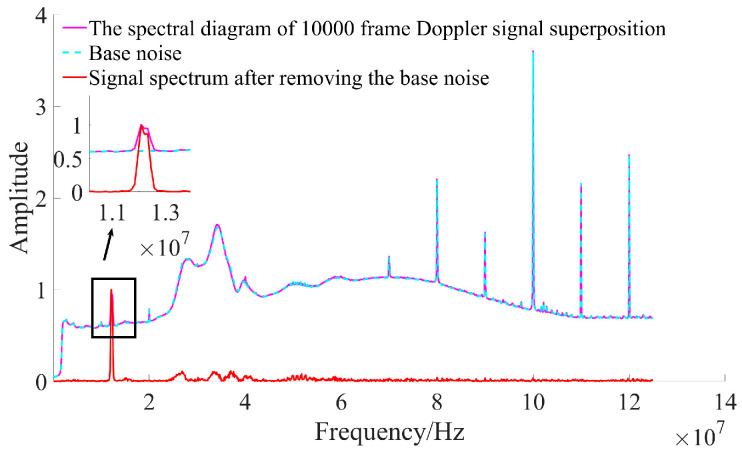
The result of removing base noise after signal spectrum superposition.

Since the interference signal depicted in Figure 10 represents the inherent noise of the system, such interference persists regardless of whether measurements are conducted. This implies that interference noise can be mitigated by first collecting a baseline noise signal and subsequently subtracting this baseline after the spectral superposition of the Doppler signals. Before the measurement begins, the movement speed is 0. At this time, the output of the system is the noise base. It is collected and superimposed in the frequency domain as the base noise. After the signal is superimposed, the base noise is removed. Figure 11 illustrates the relationship between the direct superposition of the Doppler signal spectrum and base noise, as well as the resulting signal spectrum after the removal of base noise. As shown in Figure 11, the variation trends of both baseline noise and the signal spectrum after superposition are basically consistent. Upon removal of the baseline noise, the signal peak becomes distinctly identifiable within the signal spectrum.

According to the analysis in Section 2.3, $N_{m}∼N\left(m\mu_{f},m\sigma_{f}^{2}\right)$. Since the noise of the system is subject to independent homo-distribution, the base noise has the same expectation and variance as the signal spectrum, that is, $N_{m0}∼N\left(m\mu_{f},m\sigma_{f}^{2}\right)$. From the property of Gaussian distribution:

(16) $$N_{m}-N_{m0}∼N\left(0,\sigma_{0}^{2}\right)$$

where $m\sigma_{f}^{2}=\frac{1}{2}\sigma_{0}^{2}$. Assuming N is the amplitude distribution of the signal spectrum after removing the base noise. Therefore, $N=\left|N_{m}-N_{m0}\right|$ follows a half-normal distribution, and the probability density function is:

(17) $$f_{N}\left(y\right)=\frac{\sqrt{2}}{\sigma_{0}\sqrt{\pi }}\exp\left(-\frac{y^{2}}{2\sigma_{0}^{2}}\right)$$

From the above formula, it can be concluded:

(18) $$E\left(y\right)=\sigma_{0}\sqrt{\frac{2}{\pi }}$$

(19) $$Var\left(y\right)=\sigma_{0}^{2}\left(1-\frac{2}{\pi }\right)$$

After removing the base noise, the signal peak will change from *mS* to $mS-m\mu_{f}$, so the relative prominence of the signal peak after removing the base noise is:

(20) $$RP_{m0}=\frac{y-\sqrt{\frac{2}{\pi }}\sigma_{0}}{\sqrt{1-\frac{2}{\pi }}\sigma_{0}}=\frac{mS-m\mu_{f}-\sqrt{\frac{2}{\pi }}\sqrt{2m}\sigma_{f}}{\sqrt{1-\frac{2}{\pi }}\sqrt{2m}\sigma_{f}}$$

According to Equations (15) and (20):

(21) $$\frac{RP_{m0}}{RP_{m}}=\frac{mS-m\mu_{f}-\sqrt{\frac{2}{\pi }}\sqrt{2m}\sigma_{f}}{\sqrt{1-\frac{2}{\pi }}\sqrt{2m}\sigma_{f}}\frac{\sqrt{m}\sigma_{f}}{mS-m\mu_{f}}=\frac{S-\mu_{f}-\sqrt{\frac{2}{\pi }}\sqrt{\frac{2}{m}}\sigma_{f}}{\sqrt{1-\frac{2}{\pi }}\sqrt{2}\left(S-\mu_{f}\right)}$$

When the number of superimposed frames is large enough, according to Equations (15) and (21):

(22) $$RP_{m0}\approx \frac{1}{\sqrt{1-\frac{2}{\pi }}\sqrt{2}}RP_{m}=1.173\sqrt{m}RP_{1}$$

**Figure 12:**
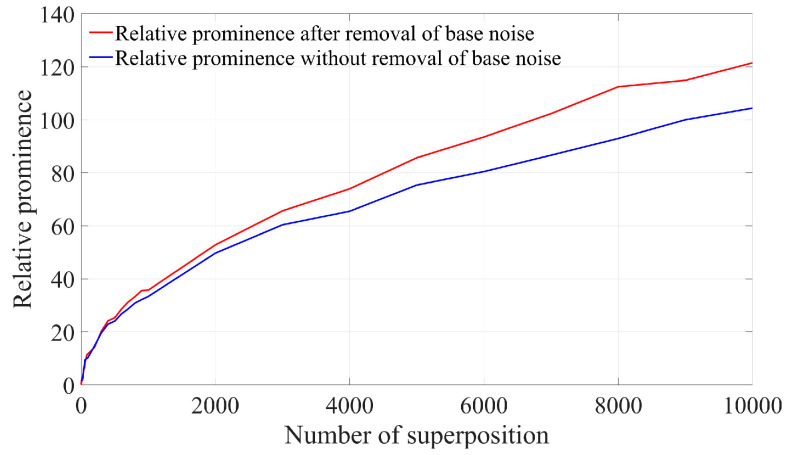
Simulation results of relative prominence change with the number of superimposed frames before and after removing the bottom noise.

As indicated by the above formula, the relative prominence of removing base noise after spectrum superposition can be increased by a factor of 1.173 compared with that without removing base noise after spectrum superposition. Consequently, the relative prominence of removing base noise after spectrum superposition is increased to times compared with that of single frame spectrum. As can be seen from Figure 12, after Doppler signal spectrum superposition, the relative prominence of removing base noise and not removing base noise is 129.05 and 107.674, respectively, which is basically consistent with the results given in Equation (22).

**Figure 13:**
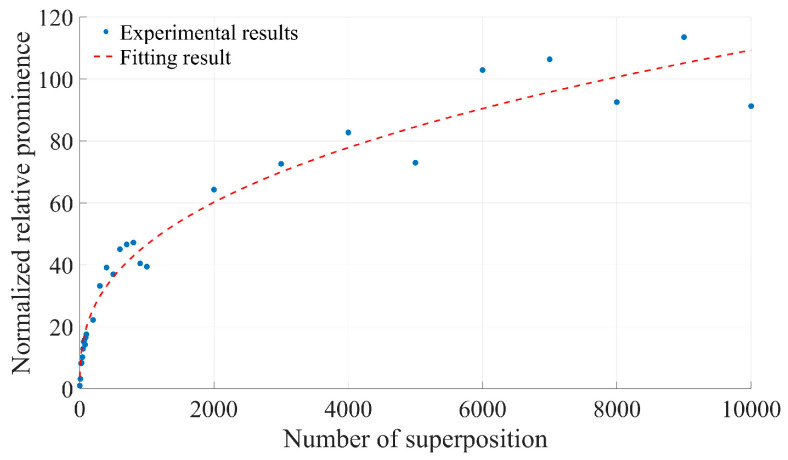
Experimental results of relative prominence change with the number of superimposed frames before and after removing the bottom noise.

Figure 13 illustrates the variation in normalized relative prominence after the superposition of the Doppler signal spectrum obtained from the experiment and the subsequent removal of baseline noise. It is evident that the relative prominence after superimposing 10,000 frames increases by approximately 109.282 times compared to that of a single-frame signal, which aligns closely with the results of the theoretical analysis.

#### 3.2.2. Selection of the Threshold of Relative Prominence

In the actual measurement process, a relative significance threshold should be set to judge whether the Doppler signal is valid. According to the analysis in Section 2.3, When the base noise is not removed, *RP_m_* follows the standard normal distribution, that is, $RP_{m}∼N\left(0,1\right)$. Set the threshold of relative prominence as *RP_T_*, then:

(23) $$P(RP_{T})=\frac{1}{\sqrt{2\pi }}e^{-\frac{RP_{T}^{2}}{2}},$$

According to the 3*σ* principles of normal distribution, when the relative prominence of the signal spectrum peak *RP* > 3, the peak has a 99.86% probability of a Doppler signal.

When the base noise is removed, according to Equation (20):

(24) $$d\left(RP_{m0}\right)=\frac{\sqrt{1-\frac{2}{\pi }}}{\left(1-\frac{2}{\pi }\right)\sigma_{0}}dy$$

By substituting Equations (20) and (24) into Equation (17), the probability distribution function of relative prominence can be obtained as follows:

(25) $$f_{N}\left(RP_{m0}\right)=\sqrt{\frac{2}{\pi }\left(1-\frac{2}{\pi }\right)}\exp\left(-\frac{1}{2}{\left(\sqrt{1-\frac{2}{\pi }}RP_{m0}+\sqrt{\frac{2}{\pi }}\right)}^{2}\right)$$

Assuming that the threshold of relative prominence is 3, according to Equation (25), the probability that the peak of this spectrum is the target signal is:

(26) $$P\left(RP_{m0}\geq 3\right)=1-\int_{3}^{+\infty }\sqrt{\frac{2}{\pi }\left(1-\frac{2}{\pi }\right)}\exp\left(-\frac{1}{2}{\left(\sqrt{1-\frac{2}{\pi }}RP_{m0}+\sqrt{\frac{2}{\pi }}\right)}^{2}\right)dRP_{m0}\approx 99.08\%$$

According to Equation (26), when the relative significance of a point on the signal spectrum is greater than 3, there is a 99.08% probability that the peak value of the Doppler signal is located at that point. It can be seen from Equations (23) and (26) that no matter how the accumulated noise changes, the value probability of *RP_T_* or *RP_mT0_* has nothing to do with it, which is similar to constant false alarm processing in radar signal processing. Therefore, although the absolute values of both signal and noise increase with the number of superimposed frames in the frequency domain, the SNR remains unchanged. However, relative prominence can still accurately reflect signal quality. In practical measurements, the threshold value can be established based on the required recognition accuracy.

#### 3.2.3. The Signal Superposition Effect at Different Speeds

**Figure 14:**
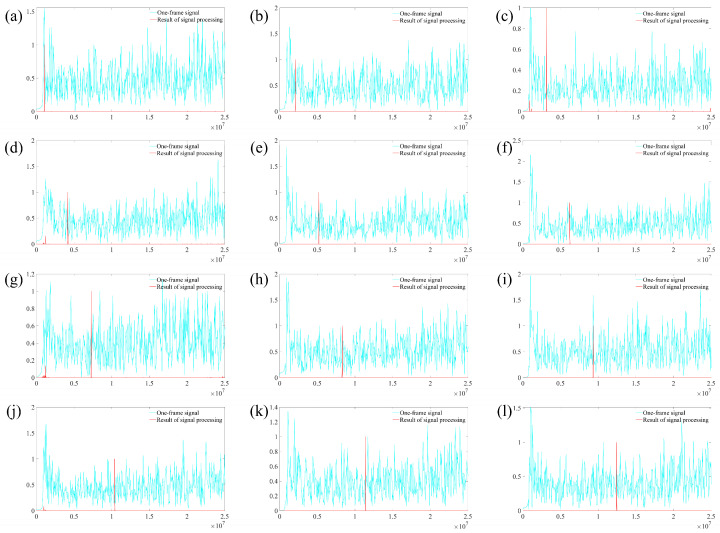
The signal spectrum at different speeds: (**a**) *w* = 100°/s; (**b**) *w* = 200°/s; (**c**) *w* = 300°/s; (**d**) *w* = 400°/s; (**e**) *w* = 500°/s; (**f**) *w* = 600°/s; (**g**) *w* = 700°/s; (**h**) *w* = 800°/s; (**i**) *w* = 900°/s; (**j**) *w* = 1000°/s; (**k**) *w* = 1100°/s; (**l**) *w* = 1200°/s.

During the experiment, the angular speed of the turntable was controlled within the range of 100°/s to 1200°/s and measured at intervals of 100°. The signal spectrum was obtained through signal superposition and by removing baseline noise. The signal spectra acquired at different rotational speeds are presented in Figure 14. In this figure, the cyan curve represents the spectrum of the single-frame Doppler signal, while the red curve corresponds to the spectrum after processing. To facilitate the comparison of the spectral amplitudes, the curves in the figure have been normalized based on the target peak value. As shown in Figure 14, the target peak cannot be distinguished from the signal spectrum without signal superposition.

**Figure 15:**
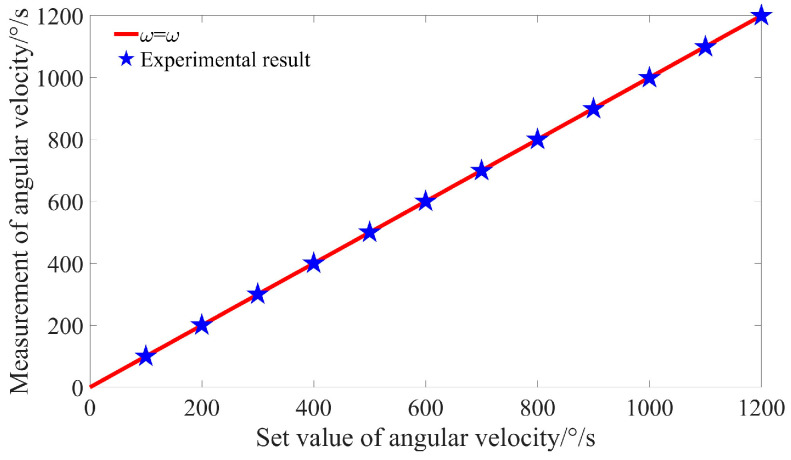
The signal spectrum at different speeds.

After applying signal superposition and baseline noise removal, the signal peak location becomes clearly identifiable. By reading the signal frequency from the spectrum depicted in Figure 14, the angular velocity of the turntable can be calculated. The relationship between the measured values and the set values at various angular velocities is illustrated in Figure 15. It is evident from the figure that the measured values obtained after signal superposition align closely with the set values.

## 4. Conclusions

This paper introduces a signal processing method for weak Doppler signals. By superimposing Doppler signals in the frequency domain and removing baseline noise, fixed-frequency noise interference can be mitigated, allowing for accurate identification of the peak values of Doppler signals. Concurrently, relative prominence is defined as a metric to evaluate the quality of these signals. A comparison between SNR and relative prominence leads to the conclusion that relative prominence more accurately represents the quality of Doppler signals. Theoretical analysis and experimental results demonstrate that m-frames Doppler signal superposition in the frequency domain can enhance relative prominence by a factor of $\sqrt{m}$, with an increase $1.173\sqrt{m}$ achievable upon removal of baseline noise. This approach also offers guidance for threshold setting based on relative prominence. When the threshold for relative prominence is established at 3, there is at least a 99.08% probability that the frequency spectrum peak corresponds to the target signal. This method holds significant potential for expanding application scenarios of LDV and enhancing its detection capabilities for weak Doppler signals.

## Data Availability

The data presented in this study are available on request from the corresponding author due to privacy.
